# Supplementary material for: Willingness to participate in, support or carry out scientific studies for benefit assessment of available medical interventions: A stakeholder survey
Source: PLoS One. 2022 Aug 12;17(8):e0271791. doi: 10.1371/journal.pone.0271791 (PMC9374247; doi:10.1371/journal.pone.0271791)
Supplement: S2 Appendix — (PDF) [file pone.0271791.s002.pdf]

S2 Appendix: Contacted associations and societies of the stakeholder groups

**Patient representatives**
**Stabstelle Patientenbeteiligung des G-BA /** Patient representative spokespersons of the G-BA
**Deutscher Behindertenrat e.V. /** The German Council of People with Disabilities
**BundesArbeitsGemeinschaft der PatientInnenstellen /** The Federal Syndicate of Patient Interest Groups
**Deutsche Arbeitsgemeinschaft Selbsthilfegruppen e.V.** / The German Syndicate of Self-Help Groups
**Verbraucherzentrale Bundesverband e.V. /** The Federation of German Consumer Organisations

**Healthcare providers**
**Marburger Bund** Verband der angestellten und beamteten Ärztinnen und Ärzte Deutschlands e.V.
**Hartmannbund** Verband der Ärzte Deutschlands e.V.
**NAV-Virchow-Bund** Verband der niedergelassenen Ärzte Deutschlands e.V.
**Verband leitender Krankenhausärzte Deutschland e.V.**
**Berufsverband Deutscher Psychologinnen und Psychologen** / The German Association of psychologists
**Deutscher Berufsverband für Pflegeberufe - Bundesverband** / The German Nurses Association
**Spitzenverband der Heilmittelverbände e.V.**

**Scientists**
**Zentrum klinischer Studien Freiburg** / The Clinical Trials Unit, University of Freiburg
**Deutsches Netzwerk Evidenzbasierte Medizin** / German Network for Evidence-based Medicine
**Deutsches Netzwerk Versorgungsforschung e.V.** / German Network for Health Services Research

**Private sector**
**VDGH Verband der Diagnostica-Industrie e.V.** / The German Diagnostics Industry Association
**BVMed Bundesverband Medizintechnologie e.V. /** The German Medical Technology Association
**SPECTARIS Deutscher Industrieverband für Optik, Photonik, Analysen- und Medizintechnik e.V. /** German Industry Association for Optics, Photonics, Analytical and Medical Technologies
**ZVEI e.V.** / Germany’s Electro and Digital Industry
**vfa. Verband forschender Pharma-Unternehmen** / German Association of Research-Based Pharmaceutical Companies
**B.A.H. Bundesverband der Arzneimittel-Hersteller e.V. /** German Medicines Manufacturers‘ Association
**BPI Bundesverband der Pharmazeutischen Industrie e.V. /** The German Pharmaceutical Industry Association
