# Supplementary material for: Willingness to participate in, support or carry out scientific studies for benefit assessment of available medical interventions: A stakeholder survey
Source: PLoS One. 2022 Aug 12;17(8):e0271791. doi: 10.1371/journal.pone.0271791 (PMC9374247; doi:10.1371/journal.pone.0271791)
Supplement: S3 Appendix — Tables A1-A6 present all general motives mentioned by the respondents of each stakeholder group for or against engagement in post-entry studies. (PDF) [file pone.0271791.s003.pdf]

S3 Appendix: General motives for engagement

Table A1 Patients' motives for and against participation

**Motives for participation**		
	number (n)	percentage (%)
Personal relevance	22	52.4
Improvement of quality of methods	22	52.4
Improvement of healthcare	20	47.6
Benefits for future patients	19	45.2
Interest	16	38.1
Recommendation	9	21.4
Advantages for own health	7	16.7
Others	6	14.3
**Motives against participation**		
	n	%
Poor integration into daily life	23	51.1
Strain and negative impact	17	37.8
Lack of trust in study personnel	15	33.3
Lack of personal relevance	7	15.6
Lack of necessity	6	13.3
Lack of benefits	5	11.1
Insufficient compensation	4	8.9
Others	9	20.0
No relevant motives	11	24.4

Others: motives with less than 10% (number); pro – financial compensation (4), integration of study tasks into daily life (2); contra – insufficient compensation (4), lack of interest (3), free answers (2)

Table A2 Patient representatives' motives for and against support

**Motives for support**		
	n	%
Improvement of healthcare	10	58.8
Interest	8	47.1
Improvement of quality of methods	8	47.1
Benefits for future patients	8	47.6
Trust in study personnel	7	41.2
Clinical relevance	5	29.4
Others	2	11.8
**Motives against support**		
	n	%
Strain for participants	6	35.3
Lack of trust in study personnel	4	23.5
Lack of necessity	4	23.5
Poor integration into daily life	4	23.5
Lack of clinical relevance	4	23.5
Others	1	5.9
No relevant motives	8	47.1

Others: motives with less than 10% (number); pro – personal relevance (1), integration of study tasks into daily life (1); contra – lack of benefits (1)

Table A3 Healthcare providers' motives for and against support

**Motives for support**		
	n	%
Improvement of healthcare	42	59.2
Benefits for future patients	36	50.7
Improvement of quality of methods	29	40.8
Personal relevance	25	35.2
Interest	24	33.8
Provision of additional data	12	16.9
Trust in study personnel	8	11.3
Others	17	23.9
**Motives against support**		
	n	%
Poor integration into daily life	33	46.5
Lack of resources	27	38.0
Lack of personal relevance	19	26.8
Strain for participants	18	25.4
Insufficient compensation	14	19.7
Lack of trust in study personnel	10	14.1
Lack of attention	8	11.3
Lack of benefits	8	11.3
Others	16	22.5
No relevant motives	11	15.5

Others: motives with less than 10% (number); pro – integration of study tasks into daily life (5), benefits for participating patients (5), financial compensation (2), attention (2), marketing (1), free answers (2); contra – lack of necessity (7), free answers (4), lack of interest (3), marketing (2)

Table A4 Scientists' motives for and against carryout

Motives for carryout		
	n	%
Improvement of quality of methods	22	47.8
Improvement of healthcare	22	47.8
Benefits for future patients	18	39.1
Interest	17	37.0
Personal relevance	16	34.8
Provision of additional data	8	17.4
Others	8	17.4
**Motives against carryout**		
	n	%
Insufficient research funds	18	43.9
Lack of relevance	13	31.7
Lack of resources	12	29.3
Limitations in methods	8	19.5
Problems in implementation	8	19.5
Lack of interest	6	14.6
Strain for patients	5	12.2
Lack of benefits	5	12.2
Insufficient access to experts	5	12.2
Others	24	58.7
No relevant motives	4	9.8

Others: motives with less than 10% (number); pro – integration of study tasks into existing structures (1), access to cooperating experts (1), further use of resources (1), benefit for participating test persons (1), financial compensation (1), free answers (2); contra – inadequate compensation (4), lack of necessity (4), insufficient access to volunteers (4), lack of attention (3), further use of resources (3), free answers (3), missing integration of study tasks into existing structures (2), negative marketing (1)

Table A5 Private sector' motives for and against support

Motives for support	n	%
Progress of own methods	6	60.0
Improvement of quality of methods	5	50.0
Improvement of healthcare	4	40.0
Marketing for method	4	40.0
Provision of additional data	3	30.0
Relevance	3	30.0
Interest	2	20.0
Benefits for future patients	1	10.0
**Motives against support**	n	%
Additional costs	10	76.9
Already sufficient evaluation	6	46.2
Lack of relevance	5	38.5
Lack of necessity	2	15.4
Lack of benefits	2	15.4
Others	3	23.1

Others: motives with less than 10% (number); contra – lack of trust in study personnel (1); negative marketing (1); lack of interest (1)

Table A6 Private sectors' motives for and against carryout

Motives for carryout	n	%
Improvement of quality of methods	2	50.0
Relevance	2	50.0
Benefits for future patients	2	50.0
Interest	1	25.0
Choice of methods	1	25.0
Improvement of healthcare	1	25.0
Progress of own methods	1	25.0
Marketing for method	1	25.0
Marketing for company	1	25.0
**Motives against carryout**	n	%
Additional costs	10	83.3
Already sufficient evaluation	8	66.7
Finance	5	66.7
Difficulties in implementation	2	25.0
Lack of necessity	2	16.7
Others	1	8.3

Others: motives with less than 10% (number); contra – lack of relevance (1), limitations in methods (1), lack of interest (1)
