# Supplementary material for: Willingness to participate in, support or carry out scientific studies for benefit assessment of available medical interventions: A stakeholder survey
Source: PLoS One. 2022 Aug 12;17(8):e0271791. doi: 10.1371/journal.pone.0271791 (PMC9374247; doi:10.1371/journal.pone.0271791)
Supplement: S4 Appendix — Tables B1-B6 present all study type-specific motives mentioned by the respondents of each stakeholder group for or against engagement in post-entry studies. (PDF) [file pone.0271791.s004.pdf]

S4 Appendix: Motives with regard to study type

Table B1 Patients' motives with regard to study type

**Randomised controlled trials**	n	%
Probable withholding of a method	17	41.5
No control over intervention allocation due to randomisation	12	29.3
Worse health outcomes expected due to different treatments	9	22.0
High burden due to additional examinations	6	14.6
Others	4	9.8
No relevant motives	16	39.0
**Observational studies**	n	%
Lack of benefit due to absence of a control group	10	24.4
High burden due to additional examinations	8	19.5
Others	6	14.6
No relevant motives	24	58.4

Others: motives with less than 10% (number); RCTs – less attention expected in control group (4); OSs – no benefit for others expected (3), no benefit for me or people close to me expected, free answer (1)

Table B2 Patient representatives' motives with regard to study type

**Randomised controlled trials**	n	%
Withholding a method from the control group	6	40.0
No control over intervention allocation due to randomisation	4	26.7
Worse health outcomes expected due to different treatments	4	26.7
High burden for participants due to additional examinations	3	20.0
Others	1	6.7
No relevant motives	5	33.3
**Observational studies**	n	%
Lack of benefit for participants expected	2	13.3
Others	2	13.3
No relevant motives	11	73.3

Others: motives with less than 10% (number); RCTs – less attention expected in control group (1); OSs – lack of benefit due to absence of control group (1), high burden for participants due to additional examinations (1)

Table B3 Healthcare providers' motives with regard to study type

**Randomised controlled trials**		
	n	%
Withholding a method from the control group	16	25.8
Worse health outcomes expected due to different treatments	12	19.4
Probable disagreement with assigned method	10	16.1
Others	23	37.1
No relevant motives	8	11.3
**Observational studies**		
	n	%
Low certainty in findings of OSs	25	40.3
Lack of benefit due to absence of control group	16	25.8
No benefit for participants expected	7	11.3
Others	7	11.3
No relevant motives	29	46.8

OS: Observational study

Others: motives with less than 10% (number); RCTs – high burden for myself/ working group/ institution (6), less attention expected in control group (5), RCTs too laborious (4), no control over intervention allocation due to randomisation (3), high burden for participants due to additional examinations (2), free answers (3); OSs – no benefit for me/ working group/ institution expected (3), free answers (4)

Table B4 Scientists' motives with regard to study type

**Randomised controlled trials**		
	n	%
High demand for time and personnel	7	17.1
Expected difficulties in recruitment of volunteers	7	17.1
High risk of discontinuation of the study expected due to lack of patients' willingness to participate	7	17.1
High risk of discontinuation of the study expected due to lack of healthcare providers' willingness to engage	7	17.1
Withholding a method from the control group	5	12.2
Others	13	31.7
No relevant motives	23	56.1
**Observational studies**		
	n	%
Low certainty in findings of OSs	19	46.3
Lack of benefit due to absence of control group	14	34.1
High demand for time and personnel	5	12.2
Others	10	24.4
No relevant motives	16	39.0

OS: Observational study

Others: motives with less than 10% (number); RCTs – high implementation costs (4), high burden for participants due to additional examinations (2), worse health outcomes expected due to different treatments (2), lack of expertise / experience (2), free answers (2), probable disagreement with assigned method (1); OSs – difficulties expected in recruitment of volunteers (3), no benefit for participants expected (2), lack of expertise / experience (2), free answers (2), high implementation costs (1)

Table B5 Private Sectors' motives with regard to study type (support)

**Randomised controlled trials**		
	n	%
Withholding method from the control group	6	50.0
Lack of relevance	6	50.0
Lack of interest	4	33.3
No influence on comparator	3	25.0
Expected lack of profitability of study	2	16.7
Others	6	50.0
**Observational studies**		
	n	%
Lack of benefit due to absence of control group	4	33.3
Confounding due to low adherence and high drop-out	4	33.3
Lack of interest	3	25.0
Expected lack of profitability of study	2	16.7
Low certainty in findings of OBSs	2	16.7
others	5	41.7
No relevant motives	1	8.3

OS: Observational study

Others: motives with less than 10% (number); RCTs – free answers (5), not enough expertise/ experience (1); OSs – free answers (4), not enough expertise/ experience (1)

Table B6 Private Sectors' motives with regard to study type (carryout)

**Randomised controlled trials**		
	n	%
High demand for time and personnel	11	91.7
Lack of relevance	9	75.0
Lack of interest	4	33.3
High risk of discontinuation of the study expected due to lack of healthcare providers' willingness to engage	3	25.0
Expected lack of profitability of study	3	25.0
High risk of discontinuation of the study expected due to lack of patients' willingness to participate	2	16.7
Withholding a method from the control group	2	16.7
Difficulties expected in recruitment of volunteers	2	16.7
Others	5	41.7
**Observational studies**		
	n	%
High demand for time and personnel	7	58.3
Lack of benefit due to absence of control group	4	33.3
Lack of interest	4	33.3
Low certainty in findings of OSs	3	25.0
Confounding due to low adherence and high drop-out	3	25.0
Others	6	50.0
No relevant motives	1	8.3

OSs: Observational study

Others: motives with less than 10% (number); RCTs – free answers (4), not enough expertise/ experience; OSs – free answers (4), expected lack of profitability of study (1), not enough expertise/ experience (1)
