# Supplementary material for: Willingness to participate in, support or carry out scientific studies for benefit assessment of available medical interventions: A stakeholder survey
Source: PLoS One. 2022 Aug 12;17(8):e0271791. doi: 10.1371/journal.pone.0271791 (PMC9374247; doi:10.1371/journal.pone.0271791)
Supplement: Attachment — Submitted filename: Response to Reviewers.docx [file pone.0271791.s006.docx]

**Response to reviewers’ comments**

**Title:** Willingness to participate in, support or carry out scientific studies for benefit assessment of available medical interventions: a stakeholder survey

**Manuscript ID:** PONE-D-21-23551

We appreciate the valuable comments made by the editor and both peer reviewers and their support in improving this manuscript. We have addressed their comments in full and revised our manuscript accordingly.

| **Reviewers comments** |  |
| --- | --- |
| **Editors comments to Author** | **Author responses** |
| When submitting your revision, we need you to address these additional requirements. | We thank you for your advice. We revised our manuscript accordingly. |
| 1. Please ensure that your manuscript meets PLOS ONE's style requirements, including those for file naming. The PLOS ONE style templates can be found at  https://journals.plos.org/plosone/s/file?id=wjVg/PLOSOne_formatting_sample_main_body.pdf and  https://journals.plos.org/plosone/s/file?id=ba62/PLOSOne_formatting_sample_title_authors_affiliations.pdf | We rechecked our manuscript against the requirements of the journal and revised it where needed. |
| 2. During the internal evaluation of your manuscript we noted that informed consent was not necessary for this study. Please could you clarify whether the IRB specifically waived the need for informed consent for the study. | Thanks for highlighting this issue. In its written statement of reasons for the vote, the ethics committee of the Albert-Ludwigs-University pointed out that it was assumed that no personal data but only anonymised data would be collected and evaluated. Accordingly, the need for informed consent was waived. We address this issue by complementing our methods section with the following information:  “*No personal but only anonymised data were collected and analysed for the purpose of this study. Therefore, no informed consent for the study was necessary according to the standards of the Ethics Committee of the Albert-Ludwigs-University Freiburg and the data protection officer.*” (*ll.136-139*) |
| 3. Thank you for stating the following in your Competing Interests section:  None   Please complete your Competing Interests on the online submission form to state any Competing Interests. If you have no competing interests, please state "The authors have declared that no competing interests exist.", as detailed online in our guide for authors at http://journals.plos.org/plosone/s/submit-now   This information should be included in your cover letter; we will change the online submission form on your behalf. | We thank you for the changes you make on our behalf with regard to the statement about competing interests. We state that the authors have declared that no competing interests exist and will make sure that this information is also included where needed during the resubmission |
| **Reviewers comments** | **Author responses** |
| **Reviewer 1:** |  |
| I have read this manuscript with much interest. This is mostly a descriptive study of participants’ willingness to participate, support or carry out scientific studies for benefit assessment of medical interventions. My comments and concerns are listed below. | Thank you for your feedback and your support in improving this manuscript. |
| 1- Where exactly was the study conducted? Was there any geographical limitation? Where participants from a specific city, region, country, etc. | Thank you for pointing out these questions. We did no limit the survey to a specific geographical area. However, since the survey was in German language and recruiting was conducted mainly through German associations and societies, we assume that respondents are German-speaking people in Germany. We added this information in the method section (subheading ‘sampling’) as follows:  “*Members of the following stakeholder groups were the target population of the survey: (i) patients or patient representatives, (ii) healthcare providers (physicians and health professionals), (iii) scientists and trialists in medical and health science, and (iv) representatives of the medical or pharmaceutical private sector (e.g. medical technology or the pharmaceutical industry). No geographical limitation was applied. However, as the questionnaire was written in German, knowledge of the German language was necessary to participate in the survey.*” *(ll.148-154)* |
| 2- The introduction is quite short and choppy. It does not lead well to the objectives of the study. | To improve this section, we have added some informative sentences and moved the second paragraph down. This resulted in a number of edits that can be seen in the accompanying revised manuscript. If you still have concerns with regard to specific aspects within the introduction, we would appreciate more details about suggested changes. |
| 3- The authors devised a 51-item questionnaire for data collection. There is no information on the validity and reliability of the questionnaire. I believe that no psychometric testing been done on the questionnaire. | Thank you for your comment. In order to provide more information on the questionnaire we added the following sentences in the method section (subheading ‘questionnaire’):  “*In the course of the systematic literature searches we did not identify any suitable, validated questionnaires for the specific purpose of our study. Therefore, we designed a de-novo self-administrated questionnaire in German language.” (ll.104-106)*  *“We did not perform psychometric testing to validate our questionnaire.” (l.122)* |
| 4- Participants: There is not much information on who the participants are (except a general statement that they are from patients, patient representatives, scientists, healthcare professionals and representatives, and private sector groups). The authors used snow-ball sampling techniques to recruit further participants, but how the primary participants were identified (e.g., from which hospital, university, etc.)? | We agree with you and we added more information about the sampling and associations we contacted. Moreover, we attached a list with details of the contacted associations and societies to the S2 Appendix.  *“A google search was performed to identify relevant associations and societies representing the target stakeholder groups in Germany. In order to recruit patients and patient representatives, the office of patient representatives in the German Federal Joint Committee (Gemeinsamer Bundesausschuss, G-BA), as well as the four organisations that are currently entitled to appoint patient representatives to the G-BA were contacted. Healthcare providers (physicians, dentists, psychologists, nurses, allied health professionals) were contacted via associations representing the interests of their respective profession. Scientists and trialists were recruited via representatives of the German network for evidence-based medicine, the German network for healthcare research and the Clinical Trials Unit of the University of Freiburg. Trade organisation for the medical technology and pharmaceutical industries were approached in order to invite representatives of the private sector. The contacted associations and societies are listed in detail in the S2 Appendix.” (ll.157-168)* |
| 5- Please revise Table 1 as: a. Please add row and column totals. b. I would suggest adding row percentage instead of column percentage. c. The last block of the table (i.e., Experience) is quite confusing. I would suggest a separate table for this section with proper title. | Thank you for this thoughtful comment. To complement this table, we have added row and column totals and moved information about experience to new created tables (Table 2A and 2B).  We did not include row percentages since several respondents identified to belong to more than one stakeholder group, the sum of row percentage does no attain 100 per cent. We added an explanation of this issue as footnote to our table. |
| **Reviewer 2:** |  |
| The authors conducted an observational study to investigate the willingness of relevant stakeholder groups to participate or engage in post-entry studies. There are, at any rate, several issues to be addressed as follows. | Thank you for your feedback and your support in improving this manuscript. |
| 1) “New, innovative interventions…”, there is an extra comma. | Thanks. We made this edit. |
| 2) Sampling was not described in sufficient details. have the authors applied inclusion and exclusion criteria? How did the author select the target organisations? | We agree with you. As described above, we added more information about the sampling and the associations we contacted (*ll.148-154; ll.157-168).* |
| 3) “The completion rate was 161 calculated using the ratio of people who had submitted the final questionnaire page and those”, have the authors compared the two groups? | We did not compare the responses of those who submitted the final questionnaire page and the non-completers. First, the completers include seven respondents that were screened out. Second, among the 46 respondents who did not submit the final page, 24 were excluded from our analysis since they did not fulfil the inclusion criteria, that is, they responded to one question only. With 22 respondents remaining (distributed among four stakeholder groups), we observe that this sample size of this subgroup is too small to conduct further post-hoc analyses to obtain meaningful results. |
| 4) “For comparison between two groups an independent t-test was 167 used for a response frequency n > 30, and the Mann-Whitney-U-Test for n < 30 162”, this reviewer understands that Excel was used for descriptive analysis but what statistical tool was used for statistical tests? | Thank you for this comment. We used Microsoft Excel for both, descriptive and analytical statistics. We rephrased the sentence in our method section as follows: “*All valid responses were tabulated, and statistical analyses were conducted using Microsoft Excel 2010.*” *(ll.185-186*) |
| 5) Have the authors checked the effect of age and gender on responses? | Thank you for raising this question. We did not carry out subgroup analyses due to the small size of the sample. We had data from a total of 154 respondents (distributed among different stakeholder groups). From a methodological point of view, we think that this sample size is not large enough for further analyses. We addressed this issue in our result section as follows:  “*We did not carry out further subgroup analyses on the effect of sociodemographic variables since the number of respondents in each stakeholder group is too small to create further representative subgroups and perform reasonable analyses*.” (*ll.272-274*) |
| 6) For reasons that might or might not be under the authors' control, the figures have a low-resolution appearance | Thank you for highlighting this issue. The figure was edited according to the journal’s requirements. In case there are inadequacies, we are happy to make changes to our figure. |
