# Supplementary material for: Willingness to participate in, support or carry out scientific studies for benefit assessment of available medical interventions: A stakeholder survey
Source: PLoS One. 2022 Aug 12;17(8):e0271791. doi: 10.1371/journal.pone.0271791 (PMC9374247; doi:10.1371/journal.pone.0271791)
Supplement: Attachment — Submitted filename: Response to Reviewers.docx [file pone.0271791.s007.docx]

**Response to reviewers’ comments**

**Title:** Willingness to participate in, support or carry out scientific studies for benefit assessment of available medical interventions: a stakeholder survey

**Manuscript ID:** PONE-D-21-23551R1

We appreciate the valuable comments made by the editor and peer reviewers and their support in improving this manuscript. We have addressed their comments in full and revised our manuscript accordingly.

| **Reviewers comments** |  |
| --- | --- |
| **Editors comments to Author** | **Author responses** |
| Please review your reference list to ensure that it is complete and correct. If you have cited papers that have been retracted, please include the rationale for doing so in the manuscript text, or remove these references and replace them with relevant current references. Any changes to the reference list should be mentioned in the rebuttal letter that accompanies your revised manuscript. If you need to cite a retracted article, indicate the article’s retracted status in the References list and also include a citation and full reference for the retraction notice. | We thank you for your advice. We rechecked our reference list and revised it where needed. We added the corrections of the articles of Sheridan et al. 2020 and Eysenbach 2012 to the reference list:  *Sheridan R, Martin-Kerry J, Hudson J, Parker A, Bower P, Knapp P. Why do patients take part in research? An overview of systematic reviews of psychosocial barriers and facilitators. Trials. 2020;21(1):840. doi: 10.1186/s13063-020-04793-2. Corrected and republished from: Trials. 2020;21(1):259. doi: 10.1186/s13063-020-4197-3.*  *Eysenbach G. Improving the quality of Web surveys: the Checklist for Reporting Results of Internet E-Surveys (CHERRIES). J Med Internet Res. 2012;14(1):e8. doi: 10.2196/jmir.2042. Corrected and republished from: J Med Internet Res. 2004;6(3):e34. doi: 10.2196/jmir.6.3.e34*. |
| **Reviewer #3:** |  |
| I have some minor observations having read the revised paper. | Thank you for your feedback and your support in improving this manuscript. |
| 1) Abstract and keywords: from the keywords and abstract, I'm not sure I would pick up that this paper has its roots in HTA, decision-making and MEA's, which is explained clearly in the Introduction. I might miss it in a title and abstract screen for a systematic review of barriers to MEAs, for example. The authors might consider some adjustments to broaden their impact; I leave that to their judgement. | Thank you for this comment. We added the following phrases to the abstract:  *“Post-entry studies are a key element in managed entry agreements and aim at generating evidence about the additional benefit of new medical interventions before reimbursement decisions are made.” (ll.20-22)*  Moreover, we added *“managed entry agreements”* to the list of keywords. |
| 2) Table 2: I found this a bit confusing - were the stakeholders asked about their experience in different units? It might help to explain in the legend. | Thank you for raising this question. The item ‘experience’ differed between stakeholder groups: We asked patients, healthcare providers and scientists about the number of participation/ *engagements* in studies of medical research, whereas for patient representatives and representatives of the private sector we asked for their *experience* in medical research in years. We added this information to the methods section as follows:  *“(iv) demographic and professional background including experience in years (for patient representatives and representatives of the private sector) or number of engagements in studies of medical research (for patients, healthcare providers and scientists)”(ll.119-122)*  Furthermore we redefined the title and added a legend to Table 2A and 2B: *Table 2A. Engagement of the respondents in medical research (in number of studies)^a^*  *Table 2B. Experience of the respondents in medical research (in years)^a^*  *^a^ Wording and scales of the item ‘experience’ differed between the respective stakeholder groups. (ll. 224-227)* |
| 3) I wasn't sure how the evidence supported that patients preferred observational studies or that healthcare providers were inexperienced - might help to point it out earlier than the Discussion or Conclusion. | Thank you for highlighting this issue. Information about the characteristics of the respondents is displayed in Table 1, 2A and B. We agree with you in highlighting some results regarding the characteristics in the text, and added the following phrases:  *“The majority of respondents were female (52.6%), aged between 35 and 54 years (46.8%), and reported to have little experience with regard to participation or engagement in medical research.” (ll. 228-230)*  Differences in the willingness to participate or engage in RCTs or observational studies are already described in the results section ‘Willingness with regard to study type’ (ll.251ff). Since significant differences were only observed in the group of the scientists, we toned down our wording in the conclusion section to the following:  *“Study type was found to be less relevant to stakeholders when considering willingness to engage in a study. However, we observed tendencies that the scientific community and healthcare providers reported a higher willingness to engage in RCTs, but patients and patient representatives reported to prefer OSs.”* *(ll. 413-416)* |
| 4) I think there's an interesting paradox in the discussion, between willingness to participate in a study on an available treatment (because they assume it's therefore OK) and preference to avoid getting placebo when there's a known treatment available (the equipoise issue). The authors could make a stronger case for (their) future work on this if they chose to; again I leave that to their judgement. | Thank you for this comment. We added the following:  “*Future research that specifies details of the comparator (active or inactive) might therefore be indicated. This could contribute to resolving the paradox between high willingness of study participation as reported in our survey and other research findings that demonstrate a reluctance of study participation in general.” (ll. 354-357)* |
